# Events related to medication errors and related factors involving nurses’ behavior to reduce medication errors in Japan: a Bayesian network modeling-based factor analysis and scenario analysis

**DOI:** 10.3352/jeehp.2024.21.12

**Published:** 2024-06-11

**Authors:** Naotaka Sugimura, Katsuhiko Ogasawara

**Affiliations:** 1 Graduate School of Health Sciences, Hokkaido University, Sapporo, Japan; 2 Graduate School of Engineering, Muroran Institute of Technology, Muroran, Japan; Hallym University, Korea

**Keywords:** Bayes theorem, Japan, Medication errors, Nursing education, Patient safety

## Abstract

**Purpose:**

This study aimed to identify the relationships between medication errors and the factors affecting nurses’ knowledge and behavior in Japan using Bayesian network modeling. It also aimed to identify important factors through scenario analysis with consideration of nursing students’ and nurses’ education regarding patient safety and medications.

**Methods:**

We used mixed methods. First, error events related to medications and related factors were qualitatively extracted from 119 actual incident reports in 2022 from the database of the Japan Council for Quality Health Care. These events and factors were then quantitatively evaluated in a flow model using Bayesian network, and a scenario analysis was conducted to estimate the posterior probabilities of events when the prior probabilities of some factors were 0%.

**Results:**

There were 10 types of events related to medication errors. A 5-layer flow model was created using Bayesian network analysis. The scenario analysis revealed that “failure to confirm the 5 rights,” “unfamiliarity with operations of medications,” “insufficient knowledge of medications,” and “assumptions and forgetfulness” were factors that were significantly associated with the occurrence of medical errors.

**Conclusion:**

This study provided an estimate of the effects of mitigating nurses’ behavioral factors that trigger medication errors. The flow model itself can also be used as an educational tool to reflect on behavior when incidents occur. It is expected that patient safety education will be recognized as a major element of nursing education worldwide and that an integrated curriculum will be developed.

## Graphical abstract

[Fig f3-jeehp-21-12]

## Introduction

### Background

Medical errors are serious problems that can affect the lives of patients. According to the World Health Organization, these errors occur in approximately one in 10 healthcare encounters, and approximately half of them can be mitigated [1]. Furthermore, medical errors are reported to be a traumatic experience, as they threaten healthcare workers’ well-being at work [2]. Preventing and reducing medical errors are very important not only for patient safety and the avoidance of medical litigation risk, but also for worker safety management. To prevent these errors, it is essential to improve the knowledge and behavior of nurses who are directly involved in patient care through patient safety education.

In particular, education on medications for patient safety is a significant priority because medications are essential for the cure and care of patients. Nurses are required to continue to acquire specialized knowledge and skills related to medications after licensure, as the treatments and medicines used in each medical specialty differ. Preventing and reducing medical errors requires basic nursing education for nursing students and clinical on-the-job training for nurses. However, patient safety education is often implicit rather than explicit in the curriculum [3], and there are fewer reports of nursing educational intervention studies than in other medical fields [4] despite meaningful differences in educational standards between developed and developing countries. Thus, patient safety education has yet to be sufficiently developed in nursing, and it is necessary to examine which aspects of patient safety education are inadequate before conducting educational intervention research.

Jin et al. [5] conducted a factor analysis of medication errors focusing on communication. They categorized the factors of each error event into organizational and conditional factors, and investigated the relationships between events and factors using a Bayesian network. They noted limitations in terms of applicability when focusing on specific communication errors. They also pointed out that the occurrence of communication errors might be lower owing to the higher degree of computerization and standardization of medical operations. Multiple factors are antecedent to medical errors, including organizational factors, the work environment, patient-related factors, and human actions [6]. The importance of factors related to the knowledge and behavior of nurses as a direct cause of errors should be further emphasized.

The framework of previous studies was applied to obtain a broader classification of medication errors in order to analyze causative events and related factors [5,6]. These findings contribute to the improvement of patient safety education.

### Objectives

This study aimed to identify the relationships between medication errors and the factors affecting nurses’ knowledge and behavior using Bayesian network modeling. It also aimed to identify important factors through scenario analysis with consideration of the nursing students’ and nurses’ education regarding patient safety and medications.

## Methods

### Ethics statement

The data in this study were obtained from an anonymized and publicly available database from the Japan Council for Quality Health Care (JQ) and did not conflict with any privacy policies [7].

### Study design

We used mixed methods. First, error events related to medications and associated factors were qualitatively extracted from actual incident reports. These events and factors were then quantitatively evaluated in a flow model using a Bayesian network, and their conditional probabilities were calculated. In addition, a scenario analysis was conducted to estimate the posterior probability of an event when the prior probability of a particular factor was 0%, meaning that the factor related to an event would have been suppressed by implementing patient safety education.

### Data sources

We used the national database of adverse medical events from JQ. This organization has been conducting various activities, such as the Project to Collect Medical Near-Miss/Adverse Event Information, to maintain public confidence in healthcare services and improve their quality [7]. A total of 1,899 Japanese medical institutions are registered in this database. The database includes national hospitals, advanced treatment hospitals, and university hospitals whose participation is required by Japanese law, and general hospitals and clinics whose participation is voluntary. Incidents that must be reported are those for which the medical facility is responsible, either when an adverse event occurs in a patient or when the organization deems it necessary to report an incident for prevention, even if there is no adverse event. The database is anonymized and published using Excel data online. The incident report is formatted to report basic information, such as the date, time, location of the incident, and the job title of the person involved; attributes related to work experience; the patient’s age and disease; the type of adverse event and degree of harm; the causes of the incident; a detailed description of the circumstances of the incident; and suggested actions to improve the incident. We included the 5,313 most recent incident reports from 2022 in this database. The selection criteria were “incidents related to medications,” “nurses were involved,” and “sufficient description of the circumstances to extract contributing factors.”

### Analysis

We classified events and extracted factors from incident reports, following the steps of root cause analysis (RCA) in a previous study [5] and using a scheme that showed multiple types of factors that contributed to incidents [6]. We asked, “Why did the incident occur?”, “What actions or circumstances of the nurse or patient triggered the incident?”, and “If the incident could have been prevented, what actions should the nurse have taken?” when extracting the factors that led to the errors. The factors were then divided into the following categories: attributional factors, system factors, conditional factors, and knowledge/behavioral factors. Within each category, the factors were named and presented using descriptive statistics.

**Fig. 1.**
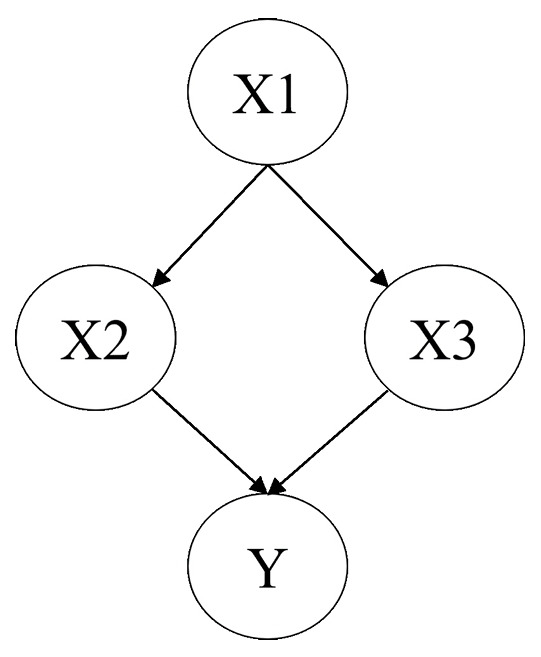
An example of Bayesian network modeling.

For Bayesian network modeling, we employed BAYOLINK ver. 7.0.1 (NTT DATA Mathematical Systems). A Bayesian network is a probabilistic model that expresses the qualitative dependence among multiple random variables using a graph structure and presents the quantitative relationship between individual variables based on their conditional probability [8]. In Bayesian network modeling, each event and factor is described as a node, and the arrow connecting them is described as an edge. As shown in Fig. 1, *X1* is the parent node of *X2*. BAYOLINK is a Java-based system developed to support Bayesian network construction [9]. This software can create Bayesian network models without coding, and it can perform inference of posterior probabilities by feeding evidence into arbitrary nodes. The analysis by BAYOLINK was performed using a greedy algorithm, and the threshold was set at 0.02. Bayesian network modeling enables the setting of whitelists and blacklists. A whitelist is a list of nodes that have a parent-child relationship, and a blacklist is a list of nodes that do not have a parent-child relationship. In this study, no whitelist was defined. Conceptually, attributional factors and system factors do not have parent nodes, so they were reflected in the blacklist for analysis. Since modeling involves cases in which completely unrelated factors are linked, the analysis was repeated by updating the blacklist as necessary until it was confirmed that the relationships between factors were consistent with the causal relationships and time series between factors. Finally, we created a flow model. This method of analysis is effective in dealing with risk assessment and decision-making under uncertainty. For example, when analyzing the relationship between medication error events and associated factors, consider the model shown in Fig. 1. *Y* is the error event and its factors are *X1*, *X2*, and *X3*. The conditional probability of *Y* is described as *P*(*Y*|*X2*, *X3*). If we assume a scenario in which factors *X2* and *X3* are controlled, that is, prior probability *P*(*X2*|*X1*) and *P*(*X3*|*X1*) are reduced, we can see the effect of the reduction on the prior probability *P*(*Y*|*X2*, *X3*).

Scenario analysis examines and evaluates various future situations and scenarios to identify their potential impacts and consequences. This method is used for risk assessment in healthcare, finance, disaster scenarios, and so forth [10]. In this study, 10 events and their associated factors were presented as a flow model. In accordance with the purpose of this study, we estimated the posterior probabilities of the events by setting the prior probabilities of nurses’ knowledge and behavioral factors to 0% in the scenario analysis. That is, we assumed that the factors of the incident would be improved by patient safety education. This analysis provided predictive value for the educational effect of actual behavioral improvements, and we could derive suggestions for prioritized content to be incorporated into patient safety education.

## Results

### Main results

**Fig. 2.**
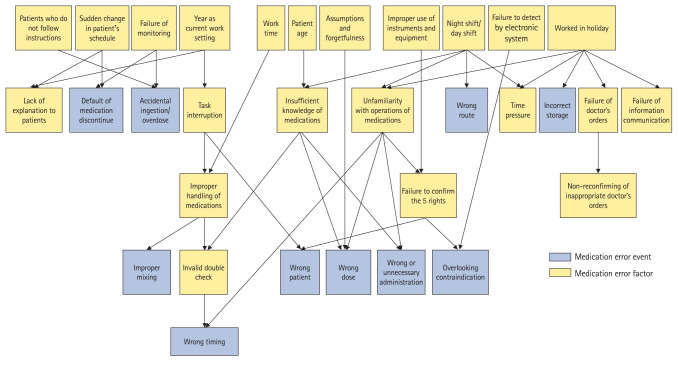
Flow model of medication errors. Values with arrows indicated conditional probabilities.

In total, 119 cases were selected (Dataset 1). Medication error events were classified into 10 categories (Table 1) and 2 attributional factors, one system factor, 8 conditional factors, and 13 knowledge and behavioral factors related to the events were extracted (Table 2). Table 3 shows the frequency of events and factors. The Bayesian network analysis revealed a flow model consisting of 5 layers (Fig. 2, Dataset 2). All events were connected to some of the factors. “Year as registered nurse” and “miscalculation” were excluded from the flow model. Table 4 shows the probability of each event when knowledge and behavioral factors related to medication error events could be mitigated (Dataset 3). Among the results, the following knowledge and behavioral factors exhibited remarkable associations with major error events: “failure to confirm the 5 rights,” “task interruption,” “improper handling of medications,” “improper use of instruments and equipment,” “insufficient knowledge of medications,” “unfamiliarity with operations of medications,” and “assumptions and forgetfulness.”

## Discussion

### Key results

This study qualitatively classified medication error events and extracted their factors from actual incident reports, and quantitatively demonstrated the relationships between events and factors in a flow model (Fig. 2). This study, which applied the framework of a previous study [5], found that rather than miscommunication-related factors, broader and more specific nurse knowledge- and behavior-level factors were related to medication errors. Furthermore, we estimated the extent to which the occurrence of these events could be prevented if these factors were controlled.

### Interpretation/comparison with previous studies

We assumed that the sample from the JQ database was representative and valid because it contained the largest number of medical institutions registered in Japan and had an appropriate incident report format. The validity of the qualitative analysis was ensured by referring to the RCA and factor schemes of previous studies [5,6]. Meanwhile, some rare events may have had an inaccurate influence on the flow model (Fig. 2). Specifically, these were “accidental ingestion/overdose,” “incorrect storage,” “wrong timing,” “failure to discontinue medication,” and “wrong route” (Table 2). The frequencies of each error event and factor were similar to those reported in a previous study [6], as dosage errors are numerous and important countermeasures have also been taken to avoid failure to confirm the 5 rights. Therefore, common issues involving human error could be latent in patient safety management in various regions and countries.

Considering the impact of rare events and focusing on the flow and scenario analysis results of major events, the following aspects need to be improved in basic nursing education and clinical on-the-job training for ensuring patient safety: “insufficient knowledge of medications,” “failure to confirm the 5 rights,” “improper use of instruments and equipment,” “task interruption,” and “unfamiliarity with operations of medications.” Since “insufficient knowledge of medications” has a strong influence on medication errors, in particular, we should not simply teach pharmacological knowledge, but also integrate knowledge of medications as part of the patient safety education curriculum, such as their handling, and practical nursing skills when administrating medications. As a bare minimum, we recommend that basic pharmacological knowledge, appropriate dosages, and the effects of high-risk medicines that are frequently used in clinical situations should be included in the basic education curriculum. Similar to surveys on the knowledge of pharmacy students [11,12], surveys on medication knowledge should be conducted among nursing students as well, and investigations and educational intervention studies should be conducted with nurses on the relationship between medication knowledge and incidents [13-15].

Regarding clinical on-the-job training, medical specialties in different wards or units use different medications. Moreover, the specialty of treatment may also affect the differences in task procedures. Although Japanese hospitals are standardized, even in Japan, the procedures and methods of treatments unique to hospital wards are in disarray, and nurses’ “unfamiliarity with operations of medications” is caused by the lack of sufficient standardization even within a single medical facility. This unfamiliarity became a factor causing medical errors when novice nurses or those who had just been transferred to a new department were involved or when new operations were introduced. In such situations, we suggest that prevention is possible through manuals, instructions, and training regarding the use of high-risk medicines in wards or newly introduced operations.

As for the “failure to confirm the 5 rights,” this is a mandatory practice. The occurrence of patient errors will be significantly reduced if this behavior is improved, as indicated by the results of the scenario analysis. As described in Table 2, with regard to “wrong patient,” most of these events occurred during oral medicine administration. As the flow model shows, “task interruption” and “improper use of supplies and equipment” were the triggers. However, “task interruption” is not unusual for nurses dealing with multiple patients. One of the root causes was “improper use of supplies and equipment,” in contexts where operations were being performed with several patients’ medications in one tray and the failure to check patients before taking their medications (Table 2). Possible countermeasures include training to make daily checking behavior habitual to avoid triggering incidents, and introducing a system to prevent human error, such as thorough patient confirmation by some devices. Appropriate staffing and management of workers’ fatigue are also effective strategies to prevent “assumptions and forgetfulness” and “task interruption.”

### Limitations

Because of the sensitivity of the analysis, we have not been able to fully examine the relationship between factors related to rare events, and it is possible that there are other factors hidden beyond those addressed in the flow model (Fig. 2).

### Suggestions

The results of this study suggest not only specific patient safety education strategies regarding medication errors, but also that the flow model itself can be used as an educational tool to reflect on behavior when incidents occur. In the future, based on the medical informatics findings of this study, it is expected that artificial intelligence systems will be developed that can more accurately predict the occurrence of medical errors and provide appropriate staffing and support to prevent them by incorporating variables such as work environment data, which provide insights into busyness, and nurses’ fatigue and performance status.

### Conclusion

Guidelines for patient safety education are scarce worldwide, and there are few reports on educational intervention studies. A Bayesian network allowed the relationships between these events and factors to be quantitatively represented in a flow model. The scenario analysis provided an estimate of the effect of mitigating nurses’ behavioral factors that trigger these incidents. The study also obtained suggestions for patient safety education in basic nursing education and clinical on-the-job training. It is expected that patient safety education will be recognized as a major element of nursing education worldwide and that an integrated curriculum will be developed.

## Figures and Tables

**Figure f3-jeehp-21-12:**
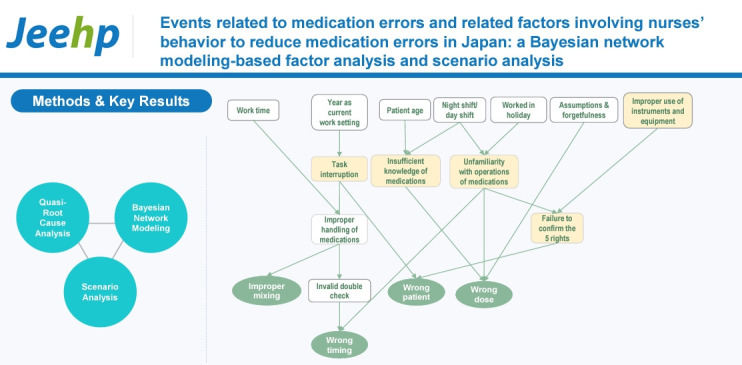


**Table 1.** Explanations of events

Event	Explanation
Wrong dose	Administered the wrong dose of medication. Most of these errors occurred in infusion situations, and these include overdosing and underdosing.
Wrong patient	Administered medicine to the wrong patient. Most of these errors were made during oral medication administration without patient verification by some devices.
Improper mixing	Performed improper mixing. Most of these errors were limited to the mixing of certain frequently used intravenous solutions.
Wrong or unnecessary administration	Administered a different medication to the one that should have been administered. Or administered a medication unnecessarily.
Overlooking contraindication	Missed contraindicated medication.
Accidental ingestion/overdose	Patient accidentally ingested or overdosed.
Incorrect storage	Stored medicine in an incorrect storage method.
Wrong timing	Administered medicine at the wrong time.
Failure to discontinue medication	Failed to discontinue medication.
Wrong route	Administered medicine by the wrong route.

**Table 2.** Explanations of factors

Factor	Explanations
Attributional factors	Factors related to the attributes of nurses who made errors.
Years as registered nurse	Divided into 5-year periods of experience as a registered nurse.
Years in current work setting	Divided into 3 stages of learning the tasks of a typical Japanese hospital ward.
<1	The stage of learning all tasks with instructions and follow-ups.
1–3	The stage of learning some tasks with instructions and follow-ups, but performing some tasks independently.
>3	The stage of performing most tasks independently.
System factor	Factors related to the organization’s systems, such as electronic medical records and educational systems.
Failure to detect by electronic system	Not equipped with the ability to detect on electronic medical record system.
Conditional factors	Factors related to conditions surrounding nurses. For example, the time of day, the type of work, and other conditions when errors occurred.
Worked in holiday	Workday was a holiday.
Work time	Work time was divided into 3 parts according to the patient’s life and the nurse’s work behavior.
Night shift/day shift	Occurred during the night shift or day shift.
Patient age	Age of patient associated with error.
Time pressure	Time pressure was present due to sudden emergencies or multiple tasks related to the patient.
Sudden change in patient’s schedule	There was a sudden change in schedule for examination or treatment.
Failure of doctor’s orders	There was a failure of doctor’s orders.
Patients who do not follow instructions	Patient did not follow the instructions of the health care professionals.
Knowledge/behavioral factors	Factors related to nurses’ knowledge state and behavior.
Failure to confirm the 5 rights	Failure to check the 5 rights.
Assumptions and forgetfulness	Assumed or forgot instructions and orders.
Invalid double check	Nurses double-checking each other was invalid.
Improper use of instruments and equipment	The use of instruments and equipment induced errors (e.g., performing tasks with multiple patients’ medications in one tray, incorrect use of equipment to administer intravenous infusions, etc.)
Insufficient knowledge of medications	Did not have sufficient knowledge.
Unfamiliarity with operations of medications	Did not know how to properly handle medications due to unfamiliarity.
Failure of information communication	Discrepancies in communication when conveying information.
Improper handling of medications	Handled medicine in the wrong way.
Failure of monitoring	Monitoring was a possibly avoidable error.
Task interruption	Task interrupted when error occurs.
Non-reconfirming of inappropriate doctor’s orders	Did not check again even though the nurse felt the doctor’s instructions were inappropriate.
Lack of explanation to patients	Not enough explanation about medication to the patient.
Miscalculation	Miscalculated appropriate medication dosage.

**Table 3.** Events and factors related to medication errors (N=119)

Node	Category	No. (%)
Event^a)^	Wrong dose	43 (36.1)
	Wrong patient	22 (18.5)
	Improper mixing	15 (12.6)
	Wrong or unnecessary administration	13 (10.9)
	Overlooking contraindication	11 (9.2)
	Accidental ingestion/overdose	7 (5.9)
	Incorrect storage	6 (5.0)
	Wrong timing	4 (3.4)
	Failure to discontinue medication	4 (3.4)
	Wrong route	2 (1.7)
Attributional factors	Year as registered nurse	
	<5	39 (32.8)
	5–9	28 (23.5)
	≥10	52 (43.7)
	Year as current work setting	
	<1	33 (27.7)
	1–3	36 (30.3)
	>3	50 (42.0)
System factor	Failure to detect by electronic system	7 (5.9)
Conditional factors	Worked in holiday	24 (20.2)
	Work time	
	9:00–16:00	54 (45.4)
	16:00–21:00	31 (26.1)
	21:00–9:00	34 (28.6)
	Night shift	
	Patient age (yr)	
	<20	
	20–59	
	≥60	
	Time pressure	42 (35.3)
	Sudden change in patient’s schedule	10 (8.4)
	Failure of doctor’s orders	10 (8.4)
	Patients who do not follow instructions	13 (10.9)
Knowledge and behavioral factors	Failure to confirm the 5 rights	59 (49.6)
	Assumptions and forgetfulness	47 (39.5)
	Invalid double check	40 (33.6)
	Improper use of instruments and equipment	35 (29.4)
	Insufficient knowledge of medications	27 (22.7)
	Unfamiliarity with operations of medications	23 (19.3)
	Failure of information communication	23 (19.3)
	Improper handling of medications	13 (10.9)
	Failure of monitoring	13 (10.9)
	Task interruption	11 (9.2)
	Non-reconfirming of inappropriate doctor’s orders	10 (8.4)
	Lack of explanation to patients	4 (3.4)
	Miscalculation	4 (3.4)

a) There were cases where multiple error events were segregated from a single incident report.

**Table 4.** Event probability when controlling knowledge and behavioral factors

Event/controlled factor^a)^	Event probability (%)	
	Posterior	Base
Wrong dose		34.9
Assumptions and forgetfulness	26.3	
Insufficient knowledge of medications	28.2	
Unfamiliarity with operations of medications	33.9	
Wrong patient		21.5
Failure to confirm the 5 rights	4.4	
Improper use of instruments and equipment	18.1	
Unfamiliarity with operations of medications	21.0	
Task interruption	18.5	
Improper mixing		15.9
Insufficient knowledge of medications	13.8	
Unfamiliarity with operations of medications	10.5	
Wrong or unnecessary administration		14.8
Improper handling of medications	5.6	
Task interruption	12.7	
Overlooking contraindication		10.6
Failure to confirm the 5 rights	6.0	
Improper use of instruments and equipment	9.7	
Unfamiliarity with operations of medications	10.4	
Accidental ingestion/overdose		6.8
Failure of monitoring	3.8	
Wrong timing		6.6
Invalid double check	3.6	
Insufficient knowledge of medications	6.3	
Improper handling of medications	6.2	
Task interruption	6.5	
Failure to discontinue medication		4.8
Failure of monitoring	3.6	

a) Prior probabilities for all controlled factors were set to 0%.

## Footnotes

**Data availability**
Data files are available from Harvard Dataverse: https://doi.org/10.7910/DVN/YCUQ8Y Dataset 1. Data file containing the original 119 incident reports.

Dataset 2. Data file containing blacklist settings in BAYOLINK.

Dataset 3. Data file containing the results of conditional probability tables.

## Supplementary materials

Supplement 1. Audio recording of the abstract.
